# Slug inhibits pancreatic cancer initiation by blocking Kras-induced acinar-ductal metaplasia

**DOI:** 10.1038/srep29133

**Published:** 2016-07-01

**Authors:** Kazumi Ebine, Christina R. Chow, Brian T. DeCant, Holly Z. Hattaway, Paul J. Grippo, Krishan Kumar, Hidayatullah G. Munshi

**Affiliations:** 1 Department of Medicine, Feinberg School of Medicine, Northwestern University, Chicago, IL 60611, USA; 2 The Robert H. Lurie Comprehensive Cancer Center of Northwestern University, Chicago, IL 60611, USA; 3 Department of Medicine, University of Illinois, Chicago, IL 60612, USA; 4 Jesse Brown VA Medical Center, Chicago, IL 60612, USA.

## Abstract

Cells in the pancreas that have undergone acinar-ductal metaplasia (ADM) can transform into premalignant cells that can eventually become cancerous. Although the epithelial-mesenchymal transition regulator Snail (Snai1) can cooperate with Kras in acinar cells to enhance ADM development, the contribution of Snail-related protein Slug (Snai2) to ADM development is not known. Thus, transgenic mice expressing Slug and Kras in acinar cells were generated. Surprisingly, Slug attenuated Kras-induced ADM development, ERK1/2 phosphorylation and proliferation. Co-expression of Slug with Kras also attenuated chronic pancreatitis-induced changes in ADM development and fibrosis. In addition, Slug attenuated TGF-α-induced acinar cell metaplasia to ductal structures and TGF-α-induced expression of ductal markers in *ex vivo* acinar explant cultures. Significantly, blocking the Rho-associated protein kinase ROCK1/2 in the *ex vivo* cultures induced expression of ductal markers and reversed the effects of Slug by inducing ductal structures. In addition, blocking ROCK1/2 activity in Slug-expressing Kras mice reversed the inhibitory effects of Slug on ADM, ERK1/2 phosphorylation, proliferation and fibrosis. Overall, these results increase our understanding of the role of Slug in ADM, an early event that can eventually lead to pancreatic cancer development.

Acinar-ductal metaplasia (ADM), a very early event in pancreatic ductal adenocarcinoma (PDAC) development, is a process by which cells lose their acinar characteristics to become duct-like in nature^1^,^2^,^3^. Since cells that have undergone ADM can transform into premalignant cells that can eventually become cancerous^1^,^2^,^3^, there is increasing interest in identifying factors that regulate these very early changes in the pancreatic tumorigenesis. There are a number of proteins expressed by acinar cells to maintain the acinar program. For example, PTF1A, important for differentiation of acinar cells from precursor cells during pancreas development, is required for maintenance of acinar cell differentiation in the adult pancreas^2^,^4^. In contrast, proteins that control the ductal state (e.g., Pdx1 and Sox9) promote the development of ADM^1^,^2^,^3^. Thus, a finely regulated balance between the acinar and ductal programs mediates ADM development.

Snail family of transcription factors, which play an important role in embryonic development, are well-known regulators of epithelial-mesenchymal transition, a process by which cells lose their epithelial characteristics to become mesenchymal^5^,^6^. We have shown that Snail (Snai1) induces invasion and scattering of pancreatic cancer cells in 3D collagen^7^. In contrast, Slug (Snai2)-expressing pancreatic cancer cells fail to scatter in 3D collagen^8^. However, inhibiting Rho-associated protein kinase ROCK1/2 reverses the effects of Slug and induces scattering of Slug-expressing pancreatic cancer cells in 3D collagen^8^. Since epithelial-mesenchymal transition can also be an early event during pancreatic cancer development^9^, we have also evaluated the effects of overexpressing Snail in acinar cells *in vivo*. We have shown that Snail overexpression in acinar cells cooperates with mutant Kras to promote ADM development^10^,^11^. Snail and Slug proteins can have similar and differing roles in development and cancer progression^5^,^6^; however, it is not known to what extent Slug mediates ADM development in the mouse pancreas.

In this report, we evaluate the role of Slug in ADM development by generating transgenic mice overexpressing human Slug in acinar cells. Slug expression in the mouse pancreas has no effect on tissue morphology. Since expression of mutant Kras in acinar cells induces ADM^10^,^12^,^13^, we evaluated the effects of co-expressing Slug and Kras in acinar cells. Surprisingly, Slug expression attenuates Kras-induced ADM development and proliferation. Co-expression of Slug with Kras also attenuates chronic pancreatitis-induced changes in ADM development and fibrosis. In addition, Slug attenuates TGF-α-induced acinar cell metaplasia to ductal structures and TGF-α-induced expression of ductal markers in *ex vivo* acinar explant cultures. Significantly, blocking ROCK1/2 activity in the *ex vivo* cultures induces expression of ductal markers and reverses the inhibitory effects of Slug by inducing ductal structures. Importantly, blocking ROCK1/2 activity in Slug-expressing Kras mice also reverses the inhibitory effects of Slug on ADM proliferation and fibrosis. Overall, these results increase our understanding of the role of Slug in ADM, an early event that can eventually lead to pancreatic cancer development.

## Results

### Slug expression in EL-Kras^G12D^ mice attenuates acinar-ductal metaplasia (ADM)

**Figure 1:**
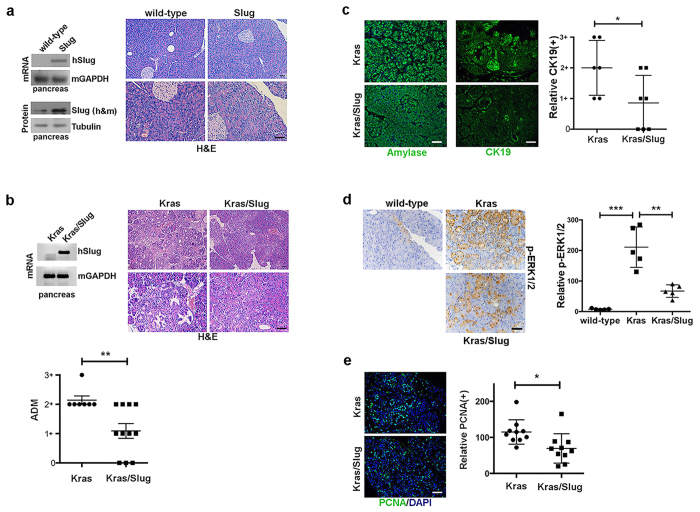
Slug expression in EL-Kras^G12D^ mice attenuates acinar-ductal metaplasia (ADM). (**a**) TRE-Slug mice were generated as detailed in the Materials and Methods and crossed with EL-tTA mice to generate Slug (EL-tTA^+^/TRE-Slug^+^) mice. The mRNA samples from pancreas of control wild-type mice and Slug mice were analyzed for human (h) Slug, and mouse (m) GAPDH by RT-PCR (top). Tissue lysates from wild-type and Slug mice were analyzed for Slug and α-tubulin by Western blotting (bottom). Sections of mouse pancreas from wild-type and Slug mice at 3-months of age were H&E stained and observed at low and high magnification by phase microscopy. (**b**) The Slug mice were crossed with EL-Kras^G12D^ mice to generate mice expressing both Slug and Kras^G12D^ in the pancreas (Kras/Slug) or littermate control mice that express only Kras^G12D^ (Kras). The mRNA samples from pancreas of control Kras mice and Kras/Slug mice were analyzed for human (h) Slug and mouse (m) GAPDH by RT-PCR. Sections of mouse pancreas from Kras and Kras/Slug mice at 3-months of age were H&E stained and observed at low and high magnification by phase microscopy. The extent of ADM was quantified as described in the Materials and Methods. (**c**) The effect of Slug expression in Kras mice on amylase and CK19 expression was determined by immunofluorescence. The extent of CK19 expression was quantified as described in the Materials and Methods. (**d**) The effect of Slug on ERK1/2 phosphorylation (p-ERK1/2) was determined by immunohistochemistry. Relative number of p-ERK1/2(+) nuclei in wild-type, Kras and Kras/Slug mice were quantified. (**e**) The effect of Slug on proliferation (PCNA) was determined by immunofluorescence. Relative number of PCNA(+) nuclei in Kras and Kras/Slug mice were quantified. Scale bars correspond to 50 μm.

Snail proteins have been shown to contribute to cancer progression^5^,^6^. We have previously published that expression of Snail (Snai1) in acinar cells of the pancreas enhances the effects of mutant Kras as demonstrated by increased ADM, increased expression of the ductal marker CK19 and increased proliferation^10^,^11^. While the role of Snail in pancreatic cancer progression is well established, the role of Slug (Snai2) in pancreatic tumorigenesis is less well understood. To evaluate the role of Slug *in vivo*, we generated TRE-Slug transgenic mice, where human Slug expression is driven by 7 tetracycline response elements (TRE) upstream of a minimal CMV promoter. The mice were crossed with EL-tTA mice to induce expression of Slug in acinar cells of the pancreas. While there was Slug mRNA and protein expression in the pancreas of EL-tTA/Slug (Slug) mice (Fig. 1a), H&E staining of Slug-expressing pancreatic tissue demonstrated no evidence of phenotypic changes (Fig. 1a). We next evaluated the effect of co-expressing Slug and Kras in the mouse pancreas. The Slug (EL-tTA/Tre-Slug) mice were crossed with EL-Kras^**G12D**^ mice to generate mice that co-express Slug and Kras in acinar cells (Kras/Slug mice). As control, we assessed mice that expressed only mutant Kras in acinar cells (Kras mice). Note that there was no change in mouse Slug expression between the wild-type mice and the Kras mice, indicating that Kras does not affect endogenous Slug expression *in vivo*. Compared to control Kras mice, Kras/Slug mice demonstrated significant attenuation of ADM development in the Kras/Slug mice (Fig. 1b). There was also less condensed amylase expression in the Kras/Slug mice compared to control Kras mice (Fig. 1c), further indicating attenuation of ADM development in the Kras/Slug mice. There was also less staining of the ductal marker CK19 in the Kras/Slug mice compared to Kras mice (Fig. 1c). Since mutant Kras can increase p-ERK1/2 levels in the mouse pancreas (Fig. 1d)^14^, we also evaluated the effect of Slug on p-ERK1/2 levels. Compared to Kras mice, Kras/Slug mice demonstrated decreased p-ERK1/2 levels (Fig. 1d). We also evaluated the effects of Slug expression on Kras-driven proliferation using PCNA as a proliferation marker. The pancreas of Kras/Slug mice demonstrated significantly reduced proliferation compared to the pancreas of Kras mice (Fig. 1e). Overall, these results indicate that Slug attenuates the effects of mutant Kras in the mouse pancreas.

### Slug expression in EL-Kras^G12D^ mice attenuates chronic pancreatitis-induced changes *in vivo*

**Figure 2:**
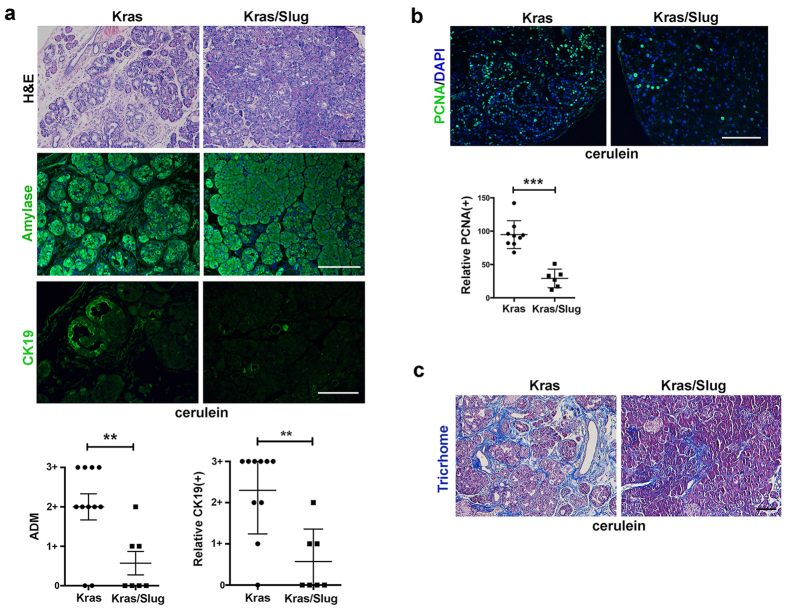
Slug expression in EL-Kras^G12D^ mice attenuates pancreatitis-induced changes *in vivo* Kras and Kras/Slug mice were generated as detailed in the Materials and Methods. Two-month old Kras and Kras/Slug mice were i.p. injected with cerulein (100 ug/kg) daily for 5 days per week for 3 weeks. Pancreatic tissue samples were collected 17 days after cessation of cerulein treatment. (**a**) The pancreatic sections were H&E stained or stained for amylase and CK19 expression by immunofluorescence. The extent of ADM and CK19 expression were quantified as described in the Materials and Methods. (**b**) The sections were stained for PCNA to assess for proliferation using DAPI to counterstain the nuclei. Relative number of PCNA(+) cells in Kras and Kras/Slug mice was quantified. (**c**) The sections were stained with trichrome (blue = fibrosis) to assess for fibrosis. Scale bars correspond to 50 μm.

Since the effects of Slug in the pancreas of EL-Kras^**G12D**^ mice were unexpected, we next evaluated the effects of Slug on ADM development following induction of pancreatitis in the EL-Kras^**G12D**^ mice. Importantly, chronic pancreatitis has been shown to contribute to pancreatic cancer development and progression^15^,^16^. Chronic pancreatitis was induced by administering cerulein, a cholecystokinin analog that causes premature release of digestive enzymes and subsequent pancreatic damage^10^,^11^. Treatment of Kras mice with cerulein for 3 weeks resulted in more pronounced ADM changes, which were attenuated in the Kras/Slug mice (Fig. 2a). There was also less condensed amylase expression in the Kras/Slug mice compared to control Kras mice (Fig. 2a). Following cerulein treatment, there was also less CK19 staining (Fig. 2a) and significantly less PCNA staining (Fig. 2b) in the pancreas of Kras/Slug mice compared to the pancreas of Kras mice. Moreover, trichrome staining showed that there was also less pancreatic fibrosis in the cerulein-treated Kras/Slug mice compared to that present in the cerulein-treated Kras mice (Fig. 2c). These results indicate that Slug can attenuate the effects of Kras even following induction of chronic pancreatitis.

### Slug attenuates TGF-α-mediated induced acinar cell metaplasia to ductal structures

**Figure 3:**
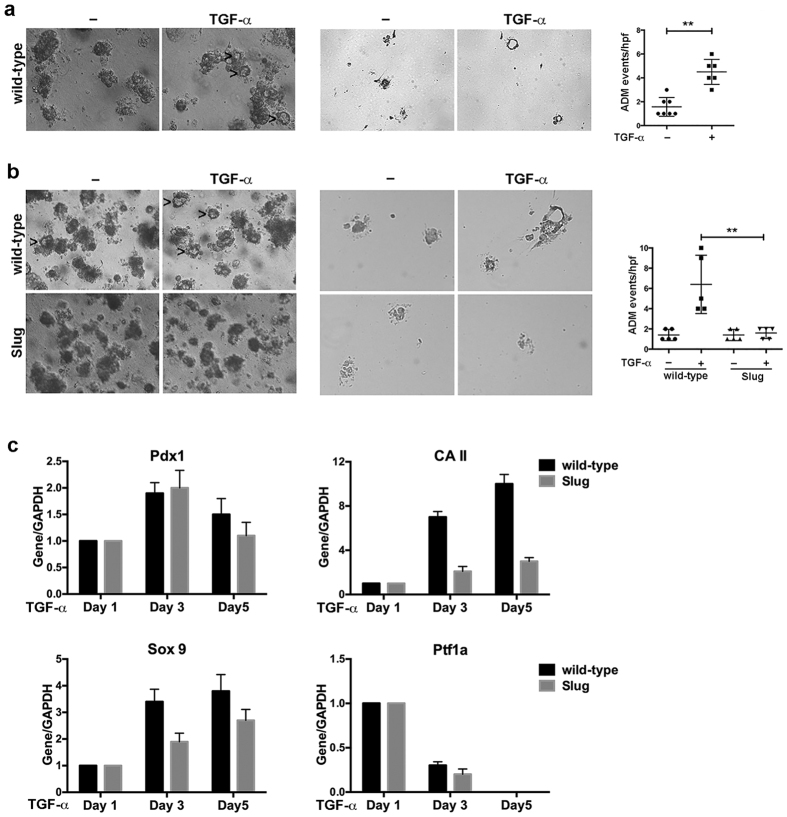
Slug attenuates TGF-α-mediated acinar cell metaplasia to ductal structures. (**a**) Primary acinar cells were isolated from wild-type B6 mice and seeded in 3D collagen. Trans-differentiation was induced with TGF-α (50 ng/ml) treatment every 2 days for 5 days. Formation of ductal structures was observed by phase microscopy (*left*), and the number of ductal structures was quantified (*right*). The collagen gels were also fixed in formaldehyde, embedded in paraffin, and gel-sections stained to identify the ductal structures (*middle*). (**b**) Primary acinar cells were isolated from wild-type and Slug mice detailed in Fig. 1, seeded in 3D collagen, and trans-differentiation induced with TGF-α (50 ng/ml) treatment every 2 days for 5 days. Formation of ductal structures was observed by phase microscopy (*left*) and by staining of sections from formaldehyde-fixed collagen gels (*middle*), and the number of ductal structures quantified (*right*). (**c**) Primary acinar cells from wild-type and Slug mice were seeded in 3D collagen and treated with TGF-α (50 ng/ml) every 2 days for 5 days to induce trans-differentiation. The mRNA samples from the primary acinar cultures were isolated after 1 day, 3 days and 5 days of treatment with TGF-α, and analyzed for ductal markers *Pdx1, carbonic anhydrase II (CA II) and Sox9* and the acinar marker *Ptf1a* using *GAPDH* as control. The relative expression in the acinar cultures was normalized to the levels present in the corresponding Day 1 acinar cultures from the respective wild-type and Slug mice. The results are representative of at least three independent experiments.

To understand how Slug attenuates ADM development in the EL-Kras^**G12D**^ mice, we cultured isolated mouse pancreatic acinar cells in 3D collagen. In this *ex vivo* explant model, acinar cells undergo ADM following treatment with growth factors that activate the EGFR^17^,^18^. Acinar cells were isolated from wild-type mice and grown in 3D collagen in the presence of TGF-α for 4–5 days. Phase microscopy of the 3D cultures showed that TGF-α treatment induced ‘duct-like’ structures in the isolated mouse acini (Fig. 3a, *left*), suggesting that acinar cells are undergoing ductal metaplasia. To further evaluate these ‘duct-like’ structures, the collagen gels were fixed, sectioned and stained. Compared to sections from untreated acinar cultures, sections from TGF-α-treated acinar cultures also showed these duct-like structures (Fig. 3a, *middle*). Quantification of these duct-like structures in the phase pictures showed that there was ~3-fold increase in the ADM events following TGF-α treatment (Fig. 3a, *right*).

We next evaluated the effects of Slug expression in these *ex vivo* explant cultures. Acinar cells were isolated from control wild-type littermates (EL-tTA+/Slug- = wild-type) and Slug-expressing mice (EL-tTA+/Slug+ = Slug), and treated with TGF-α for 5 days. Treatment of pancreatic acinar cells isolated from control littermates demonstrated a significant increase in the number of ADM events in 3D collagen (Fig. 3b). In contrast, acinar cells isolated from Slug mice showed minimal ADM events that were not affected by TGF-α treatment. These results indicate that Slug attenuates TGF-α-induced ADM development in *ex vivo* explant cultures. To understand how Slug blocks acinar cell metaplasia to ductal structures, we isolated mRNA from acinar cultures after 1 day, 3 days and 5 days of treatment with TGF-α and evaluated changes in *Pdx1* and *Sox9*, which are regulators of the ductal program and promote acinar cell metaplasia to ductal structures^19^,^20^,^21^. In addition, we evaluated the effect on *carbonic anhydrase II (CA II*), which has been shown to be up regulated in pancreatic ductal lesions^22^. While there was not a difference in the relative *Pdx1* expression between TGF-α-treated wild-type and Slug-expressing acinar cells (Fig. 3c), Slug-expressing acinar cells showed reduced relative induction of *CA II* and *Sox9* compared to the relative induction of *CA II* and *Sox9* in control wild-type acinar cells (Fig. 3c). We also evaluated whether there was activation of the acinar program in Slug-expressing mouse pancreas by evaluating expression of *Ptf1a*, the master regulator of acinar differentiation^4^. There was *decreased Ptf1a* expression in both control and Slug-expressing acinar cells at Day 3, with *Ptf1a* expression levels becoming undetectable on Day 5 of TGF-α treatment (Fig. 3c). Since Slug blocks ADM development *in vivo* and in *ex vivo* explant cultures, suppression of the ductal program rather than activation of the acinar program is likely how Slug blocks acinar cell metaplasia to ductal structures.

### Inhibition of Rho-associated protein kinases (ROCK1/2) reverses the effects of Slug in *ex vivo* 3D acinar cultures

**Figure 4:**
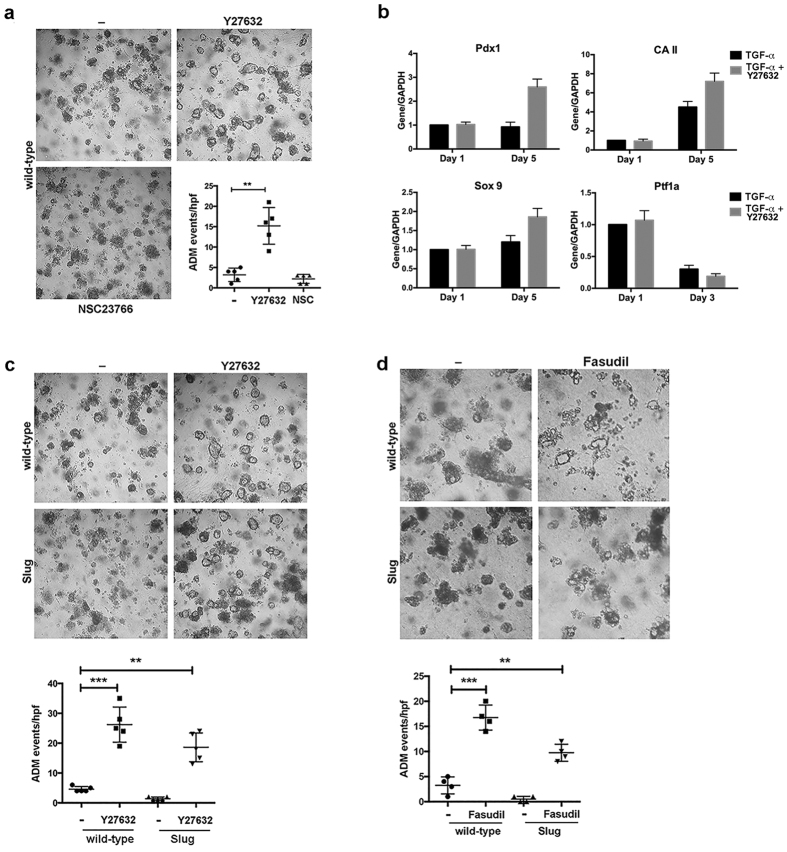
ROCK1/2 inhibition reverses the effects of Slug in 3D acinar cultures. (**a**) Primary acinar cells were isolated from wild-type B6 mice and seeded in 3D collagen. Trans-differentiation was induced with treatment with TGF-α (50 ng/ml) every 2 days for 5 days in the presence of the Rac inhibitor NSC23766 (20 μM) or the ROCK1/2 inhibitor Y27632 (10 μM). Formation of ductal structures was observed by phase microscopy and the number of ductal structures was quantified. (**b**) The primary acinar cells isolated from wild-type B6 mice were treated with TGF-α (50 ng/ml) every 2 days for 5 days and with the ROCK1/2 inhibitor Y27632 (10 μM) daily for 5 days. The mRNA samples were analyzed for ductal markers *Pdx1, carbonic anhydrase II (CA II) and Sox9* expression after 1 day or 5 days of treatment, while the acinar marker *Ptf1a* expression was analyzed after 1 day and 3 days of treatment. The relative expression was normalized to the levels present on Day 1 of the TGF-α-only treated acinar cultures from wild-type mice. (**c**,**d**) Primary acinar cells were isolated from wild-type and Slug mice detailed in Fig. 1, seeded in 3D collagen, and trans-differentiation induced with treatment with TGF-α (50 ng/ml) every 2 days for 5 days in the presence of the ROCK1/2 inhibitor Y27632 (10 μM) or the ROCK1/2 inhibitor Fasudil (25 μM). Formation of ductal structures was observed by phase microscopy and the number of ductal structures was quantified. The results are representative of at least three independent experiments.

We have previously published that the differential effects of Snail and Slug proteins on 3D collagen invasion was through their effects on Rho signaling^8^. We have demonstrated that inhibition of ROCK1/2 activity reversed the effects of Slug expression and enhanced 3D collagen invasion^8^. Thus, we examined the contribution of Rac1 and Rho signaling to Slug attenuation of ADM in *ex vivo* explant cultures. Initially, we evaluated the effects of the ROCK1/2 inhibitor Y27632 and that of NSC23766, which inhibits the interaction between Rac1 and its guanine nucleotide exchange factor Tiam1, on acinar cell metaplasia to ductal structures in *ex vivo* explant cultures^23^,^24^. Acinar cells were isolated from wild-type mice, grown in 3D collagen, treated with TGF-α and co-treated with Y27632 or NSC23766. Y27632 significantly enhanced TGF-α-induced ADM events in the 3D collagen cultures. In contrast, NSC23766 attenuated TGF-α-induced ADM events (Fig. 4a). To understand how the ROCK1/2 inhibitor Y27632 enhances ADM events, we evaluated the effects of daily Y27632 treatment on TGF-α-mediated changes in the regulators of acinar and ductal state. Daily treatment with Y27632 enhanced *Pdx1, CA II* and *Sox9* expression on Day 5. While TGF-α treatment decreased *Ptf1a* levels, daily treatment with Y27632 did not result in a significantly further decrease in *Ptf1a* levels on Day 3 (Fig. 4b). Together, these results indicate that the effects of effects of Y27632 are mediated partly through activation of the ductal program.

We next evaluated the extent to which ROCK1/2 inhibition can overcome the effects of Slug in the *ex vivo* 3D collagen cultures. Acinar cells were isolated from Slug mice and control wild-type littermates, grown in 3D collagen, and treated with Y27632. Y27632 increased ADM events in acinar cells isolated from not only the wild-type mice, but also from Slug mice (Fig. 4c). Similarly, Fasudil, another ROCK1/2 inhibitor^25^, increased ADM events in acinar cells isolated from Slug mice (Fig. 4c).

### ROCK1/2 inhibition reverses the effects of Slug expression in Kras^G12D^ mice

**Figure 5:**
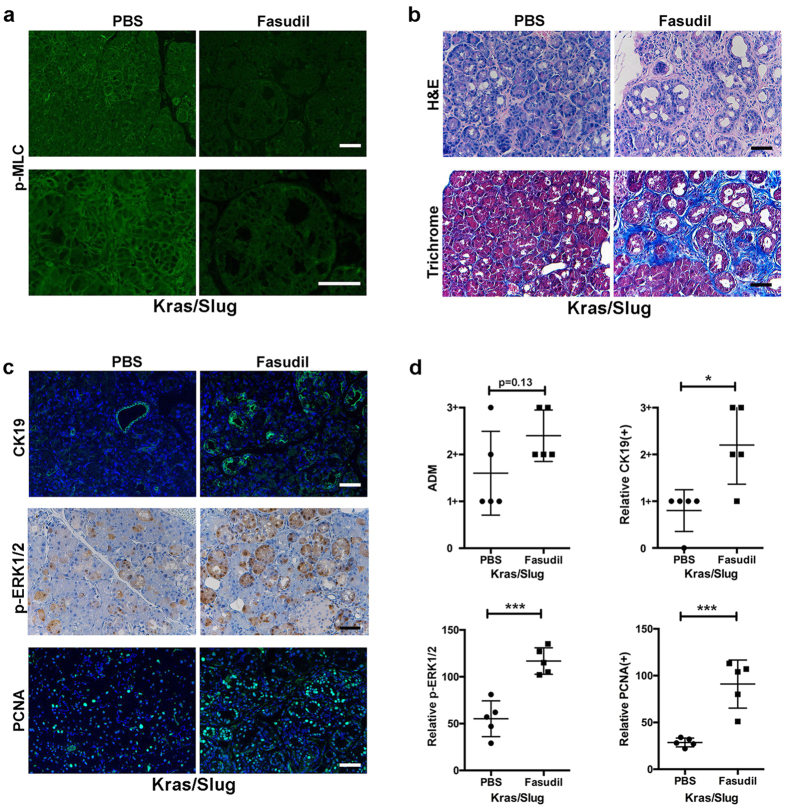
ROCK1/2 inhibition reverses the effects of Slug expression in Kras^G12D^ mice Kras/Slug mice were generated as detailed in the Materials and Methods. Two-month old Kras/Slug mice were i.p. injected with Fasudil (25 mg/kg) daily for 5 days per week for 4 weeks. (**a**) The pancreatic tissue sections were stained for p-MLC (S19), and observed at low and high magnification. (**b**) The pancreatic sections were stained with H&E to observe phenotypic changes and trichrome stained (blue = fibrosis) to assess for fibrosis. (**c**) The sections were stained for the ductal marker CK19, ERK1/2 phosphorylation (p-ERK1/2) and assessed for proliferation using PCNA. The nuclei were counterstained with DAPI. (**d**) The extent of ADM and the relative CK19 expression, ERK1/2 phosphorylation and proliferation was quantified as described in the Materials and Methods. Scale bars correspond to 50 μm.

As Fasudil has been shown to effectively block ROCK1/2 activity in mouse models^26^, we evaluated whether Fasudil could reverse the effects of Slug in EL-Kras^**G12D**^ mice. Kras/Slug mice were treated with Fasudil for 4 weeks and the pancreas were collected and analyzed for ADM events, fibrosis and proliferation. Initially, we evaluated the effects of Fasudil on p-MLC (S19). Treatment with Fasudil decreased p-MLC staining in pancreas collected from mice treated with Fasudil (Fig. 5a). Significantly, Fasudil treatment enhanced ADM events and fibrosis (Fig. 5b,d). There was also increased CK19, p-ERK1/2 and PCNA staining in pancreas from mice treated with Fasudil (Fig. 5c,d). Overall, consistent with our findings in the *ex vivo* explant cultures, ROCK1/2 inhibition also reverses the inhibitory effects of Slug on Kras-induced phenotypic changes *in vivo*.

## Discussion

Pancreatic cancer is currently the 3^rd^ leading cause of cancer related deaths in the US and it is projected to become the 2^nd^ leading cause of cancer related deaths in the US by 2020^27^,^28^. Although some progress has been made in the treatment of patients with PDAC through the use of multi-agent chemotherapy^27^,^29^, a better understanding of the factors regulating PDAC initiation may help to prevent PDAC development and/or identify potential therapeutic targets. ADM has been shown to be one of the very early changes in PDAC tumorigenesis^1^,^2^,^3^. There is compelling evidence from mouse studies demonstrating that ADM can progress to PanIN lesions, which can then further progress to PDAC tumors^1^,^2^,^3^. Importantly, ADM is frequently associated with PanIN lesions seen in human PDAC tumors^30^,^31^. In mouse studies, ADM development can be induced by expression of mutant Kras, by overexpression of TGF-α, or by induction of pancreatitis^1^,^2^,^3^. Significantly, in this report we show that Slug can attenuate both Kras-mediated and pancreatitis-induced ADM development *in vivo* and TGF-α-driven ADM development in *ex vivo* acinar explant cultures.

Snail family proteins have been shown to play an important role in pancreatic cancer development and progression^5^,^6^. We have previously shown that co-expression of Snail with mutant Kras in acinar cells potentiates the effects of Kras^10^,^11^. Snail enhances Kras-driven ADM development and associated fibro-inflammatory stromal reaction^10^,^11^. We have also shown that expression of Snail in pancreatic cancer cells promotes invasion in 3D collagen^7^,^8^. Significantly, Snail is induced early in tumorigenesis in the KPC mouse model prior to development of any obvious pancreatic tumors^9^. Even though ablation of Snail fails to prevent metastasis in the KPC mouse model, Snail mediates resistance to chemotherapy in the KPC mouse model^32^. While the role of Snail in pancreatic cancer development and progression has been demonstrated, the role of Slug in pancreatic cancer tumorigenesis is less well understood. In contrast to the effects of Snail on Kras-driven ADM development, we show that Slug attenuates Kras-driven ADM, indicating that Snail and Slug can have opposing effects during pancreatic cancer progression.

Importantly, Snail and Slug proteins were also shown to have differing roles during breast cancer development and progression^33^. Slug, but not Snail, is expressed in normal mammary epithelial cells enriched for mammary stem cells^33^. Conversely, Snail, but not Slug, is activated during breast cancer progression and is associated with the tumor-initiating phenotype in breast cancer cells^33^. Snail and Slug proteins are also found to differentially regulate pluripotency genes during reprogramming by having opposing effects on nanog promoter activity^34^. Consequently, overexpression of Snail and Slug proteins results in opposite effects on nanog-driven induction of pluripotent stem cells^34^. Moreover, Snail knockout mice die early in gestation while Slug-deficient mice are viable with minimal abnormalities^35^,^36^,^37^, indicating that Snail and Slug proteins also have differing roles during embryonic development.

Significantly, in this report we demonstrate that treatment of Kras/Slug mice with the ROCK1/2 inhibitor reverses the inhibitory effects of Slug on ADM development, proliferation and fibrosis. ROCK1/2 inhibitors also reverse the effects of Slug in *ex vivo* explant cultures and enhance ADM development. The effects of ROCK1/2 inhibitors are likely due to activation of the ductal program, as Y27632 enhances the ductal regulator *Pdx1* and *Sox9* and increases expression of the ductal marker *CA II.* Our findings are consistent with previous reports showing that Rac1 is required for mutant Kras- and pancreatitis-induced ADM development^38^,^39^. Note that Rac and Rho signaling exhibit an antagonistic relationship, and that a reciprocal balance between Rac and Rho activity determines cellular morphology and function^40^,^41^.

Overall, we show that Slug attenuates ADM development in *ex vivo* and *in vivo* models and that blocking ROCK1/2 activity can reverse the effects of Slug in these models. These results increase our understanding of the role of Slug in ADM, an early event that can eventually lead to pancreatic cancer development.

## Methods

### Antibodies/reagents

General tissue culture supplies were purchased from VWR (West Chester, PA). Antibodies against Slug (sc-15391), α-tubulin (sc-8035), amylase (SC46657) and proliferating cell nuclear antigen (PCNA; sc-56) were obtained from Santa Cruz. Antibodies against p-MLC2(S19) (3671) and p-ERK1/2(T202/Y204) (4370) were purchased from Cell Signaling, while the antibody against cytokeratin-19 (CK19; TROMA-III) was obtained from the University of Iowa. The Rac1 inhibitor NSC23766 (553508) and the ROCK1/2 inhibitor Y27632 (688001) were purchased from Calbiochem, while the ROCK1/2 inhibitor Fasudil (HA-1077) was obtained from Selleck Chemicals. Rat-tail type I collagen (354249) was purchased from Corning, collagenase I (LS004194) was from Worthington Biochemical, and cerulein (C9026) and soybean trypsin inhibitor (T6522) were obtained from Sigma.

### Transgenic mice

All animal work and procedures were approved by the Northwestern University Institutional Animal Care and Use Committee (IACUC). In addition, all experiments were performed in accordance with relevant guidelines and regulations. TRE-Slug transgenic mice, in which Slug expression is under the control of seven tet-responsive elements (TRE) upstream of a minimal cytomegalovirus (CMV) promoter, were created by the Transgenic Core Facility at Northwestern University (Chicago, IL). The TRE-Slug mice were crossed with EL-tTA mice, kindly provided by Dr. Eric Sandgren (University of Wisconsin, Madison, WI)^42^, to generate EL-tTA/TRE-Slug bigenic (Slug) mice. In EL-tTa mice, the transactivator tTa is expressed downstream of elastase promoter, thus enabling targeting of Slug to pancreatic acinar cells. The bigenic mice were further crossed with EL-Kras^G12D^ (Kras) mice^12^, which express constitutively active mutant Kras in the pancreatic acinar cells, to generate Kras/Slug mice. To evaluate the effect of chronic pancreatitis in Kras and Kras/Slug mice, two-month old mice were i.p. injected with cerulein (100 μg/kg) daily for 5 days per week for 3 weeks^10^,^11^. Pancreatic tissue samples were collected 17 days after cessation of cerulein treatment. Similarly, to evaluate the effects of ROCK1/2 inhibition *in vivo*, two-month Kras/Slug mice were i.p. injected with Fasudil (25 mg/kg) daily for 5 days per week for 4 weeks^26^.

### Histochemistry

The extent of ADM, defined by vacuolization of the normal acini with formation of abnormal ducts along with evidence of fibrosis was assessed, and a score of less than 25% (1+), 25% to 75% (2+), or more than 75% (3+) of the pancreas containing ADM was assigned to each mouse^10^,^13^. The samples were trichrome stained to assess for pancreatic fibrosis^10^,^13^.

### Immunohistochemistry/Immunofluorescence

Pancreatic tissue specimens from Kras and Kras/Slug mice were stained for amylase, CK19, PCNA, p-MLC2(S19) and p-ERK1/2(T202/Y204). Antigen retrieval was conducted as previously described^10^,^13^. Photographs for quantitative comparison were taken using FeinOptic microscope and Jenoptik ProgRes C5 camera. Number of Kras and Kras/Slug mice with no staining (0), less than 5% (1+), 5% to 25% (2+), or more than 25% (3+) of the pancreas with CK19 staining was determined. Relative number of p-ERK1/2(+) and PCNA(+) nuclei in Kras and Kras/Slug mice were quantified.

### Western blotting

Immunoblotting for Slug and α-tubulin was done as previously described^8^.

### Quantitative real time PCR

Reverse transcription of mRNA to cDNA was performed using TaqMan Reverse Transcription reagents from Applied Biosystems. Quantitative gene expression was performed with gene-specific TaqMan probes, TaqMan Universal PCR Master Mix, and the 7500 Fast Real-time PCR System from Applied Biosystems. The data were then quantified with the comparative *C*_T_ method for relative gene expression.

### *Ex vivo* 3D collagen cultures

The pancreas was removed, washed, minced into 1–2-mm pieces, and digested with solution containing collagenase I and soybean trypsin inhibitor at 37 °C for 30 min^18^,^43^. The collagen digestion was stopped by adding media containing 10% FBS. The digested pancreatic pieces were further washed and pipetted through 100-μm mesh to obtain pancreatic acinar cells. The freshly isolated pancreatic acinar cells were plated in Waymouth media solution containing neutralized collagen (2.2 mg/ml), and allowed to form 3D collagen gel over 15 min at 37 °C. The Waymouth media containing EGF (25 ng/mL) and FBS (2.5%) was then added on top of the gel. The media was replaced the next day and every other day with fresh Waymouth media containing EGF (25 ng/mL) and FBS (2.5%) that was supplemented with TGF-α (50 ng/ml). In additional experiments, the ROCK1/2 inhibitors Y276322 (10 μM) and Fasudil (25 μM), or the Rac1 inhibitor NSC23766 (20 μM) were added to the *ex vivo* cultures. After 4–5 days, the number of ‘duct-like’ structures developing in the acinar cells was counted using a Zeiss Axiovert 40 CFL microscope and pictures were taken with a Nikon Coolpix 4500 camera. The collagen gels were then fixed in formaldehyde, embedded in paraffin, and gel-sections stained to identify the ductal structures^44^. The collagen gels were also processed for gene expression analysis by qRT-PCR.

### Statistical analysis

*In vivo* and *in vitro* results were compared using *t* test analysis. Error bars represent standard error of the mean. All analyses were performed on GraphPad Prism 6 developed for Mac OS X.

## Additional Information

**How to cite this article**: Ebine, K. *et al*. Slug inhibits pancreatic cancer initiation by blocking Kras-induced acinar-ductal metaplasia. *Sci. Rep.* **6**, 29133; doi: 10.1038/srep29133 (2016).
